# Construction and validation of a notification instrument for cases of human T-cell lymphotropic virus infection: methodological study, Natal, 2025

**DOI:** 10.1590/S2237-96222026v35e20240668.en

**Published:** 2026-04-10

**Authors:** Renata Olívia Gadelha Romero, Juliana Kelly Batista da Silva, Tatiane Assone, Carolina Rosadas, Louisiana Regadas de Macedo Quinino, Carolline A Mariz, Clarice Neuenschwander Lins de Morais

**Affiliations:** 1 Escola de Saúde Pública do Rio Grande do Norte, Natal, RN, Brazil; 2 Universidade Federal da Paraíba, João Pessoa, PB, Brazil; 3 Universidade de São Paulo, Departamento de Medicina Legal, Bioética, Medicina do Trabalho e Medicina Física e Reabilitação, São Paulo, SP, Brazil; 4 Imperial College London, Department of Infectious Disease, Section of Virology, London, England; 5 Instituto Aggeu Magalhães, Fundação Oswaldo Cruz, Recife, PE, Brazil

**Keywords:** Validation Study, Deltaretrovirus Infections, Epidemiological Monitoring, Public Health, Disease Notification, Estudio de Validación, Infecciones por Deltaretrovirus, Monitoreo Epidemiológico, Salud Pública, Notificación de Enfermedades

## Abstract

**Objectives:**

To construct and validate an instrument for the notification of cases of human T-cell lymphotropic virus infection.

**Methods:**

This is a methodological study focused on content validation, conducted using the Delphi method in three sequential stages: (i) preliminary development of the instrument, based on an integrative review of the scientific literature; (ii) content validation by a committee of expert judges with recognized experience in the subject; and (iii) subsequent validation by health professionals working in healthcare services. The instruments were made available electronically. Statistical analysis of agreement among evaluators was performed using the Kappa coefficient, with agreement ≥0.75 as the minimum acceptable threshold.

**Results:**

The initial version of the instrument consisted of 58 items distributed across 10 thematic categories. Based on the expert judges’ suggestions during the two validation stages, several modifications were implemented, including reformulating certain items and adding new elements. At the end of the process, the validated instrument comprised 58 items, maintaining the same 10-category structure.

**Conclusion:**

The developed instrument demonstrated satisfactory content validity and stands out as an innovative tool for the Brazilian Unified Health System (SUS), given the absence of a standardized national form for the notification of human T-cell lymphotropic virus infection cases.

## Resumen

**Objetivos:**

Construir y validar un instrumento para la notificación de casos de infección por el virus linfotrópico de células T humanas.

**Métodos:**

Se trata de un estudio metodológico orientado a la validación de contenido, realizado mediante la técnica Delphi en tres etapas secuenciales: (i) elaboración preliminar del instrumento, fundamentada en una revisión integradora de la literatura científica; (ii) validación de contenido por un comité de expertos con reconocida experiencia en la temática; y (iii) validación posterior por profesionales de la salud que actúan en los servicios sanitarios. Los instrumentos fueron puestos a disposición por medios electrónicos. El análisis estadístico de la concordancia entre los evaluadores se realizó con base en el coeficiente Kappa, adoptando la concordancia ≥0,75 como valor mínimo de referencia.

**Resultados:**

La versión inicial del instrumento estuvo compuesta por 58 ítems distribuidos en 10 categorías temáticas. A partir de las sugerencias de los expertos participantes en las dos etapas de validación, se implementaron diversas modificaciones, lo que resultó en la reformulación de determinados ítems y en la inclusión de nuevos elementos. Al final del proceso, el instrumento validado pasó a contar con 58 ítems, manteniendo la estructura en 10 categorías.

**Conclusión:**

El instrumento desarrollado demostró una validez de contenido satisfactoria, destacándose como una herramienta innovadora para el Sistema Único de Salud, ante la inexistencia de un formulario nacional estandarizado para la notificación de casos de infección por el virus linfotrópico de células T humanas.

**Ethical aspects**

This research respected ethical principles, having obtained the following approval data:

Research ethics committee: Secretaria de Saúde do Estado da Paraíba

Opinion number: 6,690,182

Approval date: 7/3/2024

Certificate of submission for ethical appraisal: 77647323.5.0000.5186

Informed consent form: Obtained from all participants prior to data collection.

## Introduction 

Discovered in the 1980s (1), the human T-cell lymphotropic virus (HTLV) has since remained underreported and neglected by public health policies in several countries. Although little known, HTLV infection has a wide geographic distribution and, in 2012, affected between 5 and 10 million individuals worldwide. Despite the availability of these data, the lack of epidemiological studies with more up-to-date statistics may have resulted in an underestimation of the numbers (2). In Brazil, it was estimated that 1.15 million people would be infected with HTLV-1 in 2023, making the country the country with the highest absolute number of infected individuals in the world, with high prevalence rates in the North and Northeast regions (3). 

The virus is transmitted through unprotected sexual intercourse, the sharing of contaminated sharp objects, and vertical transmission, the latter being responsible for maintaining the infection across generations. Clinically, the virus may cause several manifestations that can be neoplastic, inflammatory, or infectious in nature (4), directly impacting morbidity, mortality, and the quality of life of affected individuals (5). The prevalence of infection is higher in vulnerable populations, especially those living in regions with low human development indices (6).

Recognizing its importance, HTLV-1 infection has been considered a public health problem, particularly in efforts to prevent vertical transmission (6). In this context of mobilization, Brazil has played a leading role in proposing and implementing public health policies focused on HTLV infection, including: (i) the incorporation of HTLV testing in prenatal care within the Brazilian Unified Health System (*Sistema Único de Saúde*, SUS) (6–7); (ii) the goal of eliminating vertical transmission of HTLV as a public health problem by 2030, as part of the Healthy Brazil Program (*Programa Brasil Saudável*) (8); and (iii) the inclusion of Human T-cell Lymphotropic Virus infection, HTLV infection in pregnant, parturient, or postpartum people, and children exposed to the risk of vertical transmission of HTLV on the national list of notifiable diseases (9). 

Brazil’s response to HTLV has been uneven, with states and municipalities at different stages of implementation of the recommended public policies. In some states, such as Bahia (10), Paraíba (11), Pernambuco (12), Mato Grosso do Sul, and the Federal District, and in some municipalities, such as Belo Horizonte and Rio Verde, HTLV testing is performed during prenatal care, whereas this practice is still not a reality in most states (7). 

In addition to these differing stages of implementation, the absence of a standardized national instrument for the compulsory notification of confirmed HTLV infection cases, as well as the lack of guidelines regarding the information system to be used, is noteworthy. Some states, such as Bahia (10) and Paraíba (11), have already developed their own notification instruments, integrating them into the national Notifiable Diseases Information System (*Sistema de Informação de Agravos de Notificação*, SINAN) and their respective State Monitoring Systems. However, the lack of uniformity compromises the consolidation of national data and the effectiveness of surveillance actions. It is important to emphasize that notification of HTLV infection should be carried out exclusively after diagnostic confirmation by confirmatory or molecular tests (9). 

There is a need to develop and validate a standardized nationwide notification instrument that encompasses different life stages—including children, pregnant people, postpartum people, and adults—and is applicable across public and private health services. The development of such an instrument is essential to strengthen epidemiological surveillance of HTLV infection and to guide systematic adoption of control and prevention measures. This study aimed to develop and validate an instrument to notify HTLV infection cases.

## Methods 

### Study design

This is a methodological study aimed at developing and validating a reliable, replicable instrument for the notification of cases of human T-cell lymphotropic virus infection. This type of study focuses on creating methodological tools for data collection, organization, and analysis, with a view to future application in similar contexts (13). 

### Setting 

Content validation constituted a fundamental stage in the construction of the instrument, based on the analysis of agreement regarding the items and representative domains of the instrument, according to the consensual evaluation of the experts (14). The study was conducted in three sequential stages: (i) development of the instrument, based on an integrative literature review; (ii) content validation by expert judges with recognized experience in the HTLV field; and (iii) content validation by health professionals with experience in epidemiological surveillance and in the management of sexually transmitted infections.

The development of the instrument was based on the results of the literature review, aimed at identifying scientific evidence to support its construction. For content validation, the Delphi method was applied using questionnaires sent to a group of experts, to reach consensus regarding the relevance, clarity, and importance of the instrument’s items (15). Consensus was achieved after a thorough analysis of the experts’ opinions and justifications, conducted over successive rounds of evaluation (15–16).

### Participants 

Content validation consisted of two stages. Each stage included two rounds, allowing for a more refined assessment of the modifications suggested by the judges. The first stage involved seven expert judges, all of whom had scientific expertise in HTLV. The eligibility criteria for these judges were: (i) having a *lato sensu* specialization; and (ii) having at least one year of scientific experience with HTLV. The recruitment of judges was conducted through convenience sampling via a WhatsApp group composed of specialists in the field.

The second stage of content validation involved six health professionals with work experience in sexually transmitted infection programs and in epidemiological surveillance at the municipal or state level. The eligibility criteria for these judges were: (i) professional activity in state or municipal sexually transmitted infection programs or in municipal and state epidemiological monitoring; and (ii) at least one year of professional experience in these services. These specialists were recruited via email from an institutional list provided by the Ministry of Health.

In both stages, the informed consent form was made available via email. After agreeing to participate voluntarily in the study, a second email was sent with a link to the instrument formatted in Google Forms, the notification form, and a matrix detailing the purposes and descriptions of the items, both in PDF format. A 10-day deadline was set for each judge to complete the instrument’s content validation.

### Variables 

Originally, the instrument consisted of 58 items distributed across 10 categories: general data (items 1–9); sociodemographic data (items 10–30); probable mode of transmission (items 31–33); pregnant/postpartum women (items 34–44); exposed child (items 45–48); laboratory data (items 49–50); symptoms/diseases/infections (items 51–52); disease progression (items 53–54); observations (item 55); and notifier (items 56–58). 

A five-point Likert scale was provided for the evaluation of the instrument’s items: 1) strongly disagree, 2) disagree, 3) neither agree nor disagree, 4) agree, and 5) strongly agree.

### Data sources/measurement 

Study data were collected between February and May 2024 through a questionnaire hosted on Google Forms. Subsequently, the data were entered and analyzed in an Excel spreadsheet. 

### Statistical methods

Descriptive statistics were employed, with calculations of absolute and percentage values for continuous and categorical data, in order to analyze the sociodemographic variables of the participating health professionals and expert judges. The Delphi method was used to validate the instrument’s items and domains. For statistical analysis, the Kappa coefficient was used, with values≥ 0.75 considered satisfactory for evaluating the instrument’s items and domains. The Kappa coefficient, content validity index, and universal agreement probability were used to calculate these indices (17). The Statistical Package for the Social Sciences (SPSS) software, version 26.0, was used for statistical analyses.

## Results 

In the first stage of content validation, 60 expert judges were invited, of whom 10 agreed to participate in the study’s first round. In the second round, seven expert judges who had contributed in the first round provided feedback on the instrument’s validation. For the second stage of content validation, 42 health professionals were invited, of whom 10 agreed to participate in the first round; six subsequently contributed to the second round of instrument validation. A questionnaire was developed to characterize the study participants (Table 1). 

**Table 1:** Absolute numbers (n) and percentages (%) of sociodemographic variables of the health professionals and expert judges participating in the study. Natal, 2024 (n=13)

Variables	Health professionals				Experts			
	First round		Second round		First round		Second round	
	n		n	%	n	%	n	%
**Age group** (years)								
<30	0		0	0.0	2	20.0	2	28.6
31–40	4		2	33.3	2	20.0	2	28.6
41–50	5		4	66.7	1	10.0	1	14.3
>50	1		0	0.0	5	50.0	2	28.6
Sex								
Male	2		2	33.3	3	30.0	1	14.2
Female	8		4	66.6	7	70.0	6	85.7
Profession								
Nurse	8		5	83.3	0	0.0	0	0.0
Infectious disease physician	1		0	0.0	0	0.0	0	0.0
Municipal public servant	1		1	16.6	0	0.0	0	0.0
Physician, lecturer, researcher	0		0	0.0	3	30.0	2	28.5
Graduate student	0		0	0.0	2	20.0	1	28.5
Researcher	0		0	0.0	1	10.0	1	14.2
Professor/Lecturer	0		0	0.0	3	3.0	2	28.5
**Professional experience in the current position** (years)								
Infectious disease outpatient clinic	1		0	0.0	0	0.0	0	0.0
Sexually transmitted infections program	6		5	83.3	0	0.0	0	0.0
Epidemiological monitoring	3		1	16.6	1	10.0	0	0.0
Clinical practice and teaching	0		0	0.0	5	50.0	3	42.8
Clinical practice, teaching, and research	0		0	0.0	2	20.0	2	28.5
Teaching and research	0		0	0.0	2	20.0	2	28.5
**Professional experience with HTLV^a^** (years)								
1–5	0		0	0.0	2	20.0	2	28.6
6–10	0		0	0.0	2	20.0	2	28.6
>10	0		0	0.0	6	60.0	3	42.9
**Region of professional practice in Brazil**								
North	3		2	28.5	2	20.0	2	28.5
Northeast	5		2	58.5	5	50.0	3	42.8
Central-West	1		1	14.2	0	0.0	0	0.0
South	1		1	14.2	0	0.0	0	0.0
Southeast	0		0	0.0	3	30.0	2	28.5
**Academic degree**								
Specialization	5		3	50.0	0	0.0	0	0.0
Master’s degree	5		3	50.0	4	40.0	3	42.8
Doctorate (PhD)	0		0	0.0	3	30.0	3	42.8
Postdoctoral degree (Postdoc)	0		0	0.0	3	30.0	1	14.2
**Has publications on HTLV in indexed journals**								
Yes	0		0	0.0	7	70.0	2	71.4
No	0		0	0.0	3	30.0	5	28.5
**Has previously participated in HTLV-related training** (lectures, conferences, workshops, research)								
Yes	7		3	50.0	0	-^b^	0	-^b^
No	3		2	33.3	0	-^b^	0	-^b^
Does not remember	0		1	16.6	0	-^b^	0	-^b^
**Was aware of the inclusion of HTLV infection on the national list of notifiable diseases, conditions, and public health events in public and private health services**								
Yes	9		6	100.0	0	-^b^	0	-^b^
No	1		0	0.0	0	-^b^	0	-^b^
**Is the HTLV care pathway implemented in your current service?**								
Yes	1		1	16.6	0	-^b^	0	-^b^
No	6		3	50.0	0	-^b^	0	-^b^
In progress	3		2	33.3	0	-^b^	0	-^b^

^a^HTLV – human T-cell lymphotropic virus; ^b^Items 9, 10, and 11 were specifically directed to the health professional judges, as they address issues related to HTLV in the context of health services.

The original version of the instrument was modified in accordance with the expert judges’ suggestions, which the researchers accepted. In the *sociodemographic data* domain, the option “other” was added to item 14 (sexual orientation). The probable *mode of transmission* domain underwent modifications: the name of the item was changed to “blood transmission,” and the options “use of injectable drugs,” “treatment/blood transfusion of blood derivatives,” “blood transfusion,” “occupational exposure to blood and biological materials through accidents with sharp instruments,” and “organ and/or bone marrow transplantation” were added.

Within the *sociodemographic domain*, item 19 (individual taxpayer registry number) was suggested and validated only by the health professional judges and was therefore not validated by the expert judges.

In the *exposed child* domain, item 46 (breastfeeding) was modified to include the following options: “exclusive breastfeeding,” “predominant breastfeeding,” “complementary breastfeeding,” “mixed breastfeeding,” “milk bank feeding,” “feeding by another person,” and “artificial feeding.” Item 47, “duration of breastfeeding,” was also added, with the options “less than or equal to 3 months” and “greater than 3 months.” In item 48, the collection of biological material (blood) for polymerase chain reaction testing was identified.

In the *laboratory data domain*, item 49 (laboratory evidence of HTLV infection) had the options “positive” and “negative” removed. The title of item 50 (subtype typing) was replaced with “virus type identification.” In option 3, the slash separating the identification of HTLV-1/2 types was replaced by the conjunction “and” (HTLV 1 and 2).

The symptoms/diseases/infections domain in item 52 (associated diseases/infections) was modified to include an option to specify which associated diseases or infections were identified at the time of diagnosis. The options “adult T-cell leukemia and lymphoma” were combined into a single alternative. An “other” option was also added to allow reporting of additional diseases or infections.

In the *disease progression* domain, item 53 (case progression) had all its response options modified to: “without disability,” “with disability,” “death attributable to HTLV-associated morbidity,” “death due to other causes,” “hospitalization attributable to HTLV-associated morbidity,” “hospitalization due to other causes,” and “unknown.” 

Although item 53 (progression) had a Kappa coefficient of 0.67, below the established threshold (≥0.75), the authors considered its relevance for monitoring HTLV infection cases sufficient to justify its retention in the instrument.

After the modifications suggested by the expert judges, the instrument was made available for the second stage of validation with the health professional judges, who proposed the following changes. In the sociodemographic data domain, the items “patient’s individual taxpayer registry number,” “health region,” and “sanitary district” were added. In the *pregnant/parturient/postpartum people* domain, the item “pregnant woman’s identification number in the Prenatal Follow-up System” was removed, and item 49 (laboratory evidence of HTLV infection) was renamed “laboratory evidence of confirmatory diagnosis of HTLV infection.”

In the symptoms/disease/infection domain, the option “presence of sexually transmitted infections” was added. Qual (is)”. In the original instrument, this information was located in the *sociodemographic data* domain, specifically under the item “special populations (diagnosis of sexually transmitted infections).”

After the two stages of content validation, with statistical analyses indicating agreement with the adopted reference value (Kappa coefficient ≥0.75), the instrument was finalized with 58 items, distributed across 10 categories: general data (1–9); sociodemographic data (10–30); probable mode of transmission (31–33); pregnant/postpartum women (34–44); exposed child (45–48); laboratory data (49–50); symptoms/diseases/infections (51–52); disease progression (53–54); observation (55); and notifier (56–58) (Table 2).

**Table 2:** Kappa coefficients (k) of the items and domains of the validated instrument for notification of human T-cell lymphotropic virus (HTLV) infection cases. Natal, 2024 (n=62)

Questions	Health professionals		Experts	
	Item	Domain	Item	Domain
	k	k	k	k
**General data**				
Type of notification	0.92		1.00	
Disease	1.00		0.95	
Date of notification	1.00		1.00	
Notification number	1.00		0.90	
State of notification	1.00	0.98	1.00	0.96
Municipality of notification	1.00		1.00	
Reporting health center	1.00		1.00	
National Health Establishment Registry (CNES)	0.92		0.83	
Date of diagnosis	1.00		1.00	
**Sociodemographic data**				
Patient’s name	1.00		0.92	
Date of birth	1.00		1.00	
Age	1.00		1.00	
Biological sex	1.00		0.95	
Sexual orientation	0.83		0.75	
Pregnant person	1.00		0.90	
Race/skin color	1.00		0.95	
Education level	1.00		0.95	
Brazilian Unified Health System (SUS) card number	0.90		0.95	
Individual Taxpayer Registry (CPF)	1.00		0.00^a^	
Mother’s name	1.00		0.83	
State of residence	1.00		0.95	
Municipality of residence	1.00	0.97	0.95	0.87
Address	1.00		0.82	
District	1.00		0.82	
Postal code	0.95		0.82	
Phone number	1.00		0.82	
Area	0.95		0.95	
Country	1.00		0.95	
Occupation	1.00		0.83	
Receives any government benefit	0.82		0.90	
**Probable mode of transmission**				
Vertical transmission	0.95		0.95	
Sexual transmission	0.95	0.95	0.90	0.87
Blood transmission	0.95		0.75	
**Pregnant/postpartum person**				
Has attended/is attending prenatal care	0.87		0.83	
State where prenatal care was provided	0.92		0.95	
Municipality where prenatal care was provided	1.00		0.95	
Health center where prenatal care was provided	1.00		0.95	
Laboratory evidence of HTLV	1.00		0.82	
State where delivery took place	1.00	0,93	0.90	0,92
Municipality where delivery took place	1.00		0.95	
Health service/place where delivery took place	1.00		0.95	
Date of delivery	1.00		0.95	
Type of delivery	1.00		0.90	
Pregnancy outcome	1.00		0.95	
**Exposed child**				
Certificate of live birth number	0.92		0.95	
Breastfeeding	0.87		0.90	
Duration of breastfeeding	0.92	0.92	0.75	0.83
Blood sample collected for PCR testing^b^	0.92		0.75	
**Laboratory data**				
Laboratory evidence of HTLV infection	0.87	0.94	0.83	0.81
Virus type identification	1.00		0.75	
**Symptoms/diseases/infections**				
Symptoms	0.92	0.96	0.90	0.84
Associated diseases/infections	1.00		0.75	
**Progression**				
Case progression	0.95	0.97	0.67^c^	0.84
Date of death	1.00		0.95	
Observations				
Observations field	0.95	0.95	0.95	0.95
**Reporter**				
Reporter’s name	1.00		0.95	
Position	1.00	1.00	0.95	0.93
Signature	1.00		0.90	

^a^The Individual Taxpayer Registry (*Cadastro de Pessoa Física*, CPF) item was suggested by the health professional judges after the round with the expert judges had been completed; ^b^PCR – polymerase chain reaction; ^c^By decision of the authors, this item was retained in the questionnaire.

## Discussion 

The results demonstrated that the employed methodology was effective in developing and validating the instrument’s content, as evidenced by high levels of agreement among the judges (Kappa coefficient ≥ 0.75). The validation process allowed for adjustments to different domains of the instrument, particularly those related to sociodemographic data, mode of transmission, child exposure, laboratory data, and clinical progression. The final version of the instrument consisted of 58 items distributed across 10 domains.

The limited number of expert judges who participated in the instrument’s validation stages was a limitation of this study. This was mainly due to the scarcity of professionals with well-established expertise in HTLV, an infection that remains neglected in public health. However, the judges’ technical qualifications ensured the reliability and depth of their contributions. 

Another limitation concerned the absence of semantic analysis of the instrument among professionals responsible for case notification, an important step to ensure the clarity and comprehensibility of the proposed items and domains. This phase could not be carried out due to operational constraints and limited time. 

Nevertheless, the wording of the items and domains was carefully developed, using clear, accessible, and appropriate language for the notification of HTLV infection, supported by scientific literature and the contributions of the participating judges. The proposed instrument does not encompass the full scope of HTLV infection notification and was not intended to be definitive. Therefore, the instrument remains open to review and adaptation in line with new scientific evidence and data demands that may arise as the response to HTLV evolves.

The modification proposed in the *sociodemographic data* domain, particularly in the item “sexual orientation,” was made considering sexual behaviors associated with greater vulnerability to HTLV-1 and HTLV-2 infection, particularly regarding sexual transmission (18).

The prevalence of HTLV-1 and HTLV-2 infection in specific groups—such as men who have sex with men, transgender people, and sex workers—remained high in 2021 (19–20). The suggested reformulations in the *probable mode of transmission* domain were supported by evidence, particularly regarding the inclusion of “use of injectable drugs,” “treatment/blood transfusion of blood derivatives,” “blood transfusion,” “occupational exposure to blood and biological materials through accidents with sharp instruments,” and “organ and/or bone marrow transplantation” (19–20).

The reformulation of the *exposed child* domain was also noteworthy, especially concerning breastfeeding (item 46 and the addition of item 47 on breastfeeding duration). Breastfeeding is one of the main factors contributing to vertical transmission of HTLV. Evidence has shown that the longer the duration of breastfeeding, the higher the risk of transmission. However, breastfeeding for more than three months already increases the risk of virus transmission (21–23). 

Still within the *exposed child* domain, the inclusion of polymerase chain reaction (PCR) testing as item 48 was suggested. Among genome amplification–based methods, PCR stands out with a sensitivity of approximately 70% and specificity of 100%, and it is less costly than the most commonly used confirmatory serological test, the Western blot (24–26).

In the symptoms/diseases/infections domain, modifications were made to item 52 (associated diseases/infections). In the disease progression domain, item 53 (case progression) was reformulated. Although most people living with HTLV remain asymptomatic, some individuals develop diseases associated with the infection that, in general, present serious morbidities and mortality (27). 

HTLV-1 infection early in life is a risk factor for the development of adult T-cell leukemia/lymphoma, with an estimated disease development probability of 7% in men and 3% in women. Based on the life expectancy of 75 years in Brazil, it is estimated that between 427 and 1,333 cases of adult T-cell leukemia/lymphoma occur annually (28).

The creation of this notification instrument represents an innovative, timely, and strategic contribution to strengthening epidemiological surveillance of HTLV infection, especially given its inclusion on the national list of notifiable diseases. Its use may support more effective public health policies, promote better resource allocation, and enhance understanding of the true magnitude of this condition in the country.

## Data Availability

The research data (tables, the developed instrument, and the database) are available in the SciELO Data repository (29) and on the eduCapes portal (30).
