# Supplementary material for: Construction and validation of a notification instrument for cases of human T-cell lymphotropic virus infection: methodological study, Natal, 2025
Source: Epidemiol Serv Saude. 2026 Apr 10;35:e20240668. doi: 10.1590/S2237-96222026v35e20240668.en (PMC13078672; doi:10.1590/S2237-96222026v35e20240668.en)
Supplement: Translation

# Translation: Construção e validação de instrumento de notificação de casos de infecção do vírus linfotrópico de células T humanas: estudo metodológico, Natal, 2025

**DOI:** 10.1590/S2237-96222026v35e20240668.pt

**Authors:** Renata Olívia Gadelha Romero, Juliana Kelly Batista da Silva, Tatiane Assone, Carolina Rosadas, Louisiana Regadas de Macedo Quinino, Carolline A Mariz, Clarice Neuenschwander Lins de Morais

**Affiliations:** 1 Escola de Saúde Pública do Rio Grande do Norte, Natal, RN, Brasil; 2 Universidade Federal da Paraíba, João Pessoa, PB, Brasil; 3 Universidade de São Paulo, Departamento de Medicina Legal, Bioética, Medicina do Trabalho e Medicina Física e Reabilitação, São Paulo, SP, Brasil; 4 Imperial College London, Department of Infectious Disease, Londres, Inglaterra; 5 Instituto Aggeu Magalhães, Fundação Oswaldo Cruz, Recife, PE, Brasil

**Keywords:** Estudo de Validação, Infecções por Deltaretrovírus, Vigilância Epidemiológica, Saúde Pública, Notificação de Doenças

## Abstract

**Objetivos:**

Construir e validar instrumento para a notificação de casos da infecção pelo vírus linfotrópico de células T humanas.

**Métodos:**

Trata-se de estudo metodológico voltado à validação de conteúdo, conduzido por meio da técnica Delphi em três etapas sequenciais: (i) elaboração preliminar do instrumento, fundamentada em revisão integrativa da literatura científica; (ii) validação de conteúdo por um comitê de juízes especialistas com reconhecida experiência na temática; e (iii) validação subsequente por profissionais de saúde atuantes nos serviços de saúde. Os instrumentos foram disponibilizados por meio eletrônico. A análise estatística da concordância entre os avaliadores foi realizada com base no coeficiente Kappa, adotando-se a concordância ≥0,75 como valor de referência mínimo.

**Resultados:**

A versão inicial do instrumento foi composta por 58 itens distribuídos em 10 categorias temáticas. A partir das sugestões dos juízes participantes nas duas etapas de validação, diversas modificações foram implementadas, o que resultou na reformulação de determinados itens e na inclusão de novos elementos. Ao final do processo, o instrumento validado passou a contar com 58 itens, mantendo-se a estrutura em 10 categorias.

**Conclusão:**

O instrumento desenvolvido demonstrou validade de conteúdo satisfatória, destacando-se como ferramenta inovadora para o Sistema Único de Saúde, diante da inexistência de formulário nacional padronizado para a notificação de casos de infecção pelo vírus linfotrópico de células T humanas.

**Aspectos éticos**

Esta pesquisa respeitou os princípios éticos, obtendo os seguintes dados de aprovação: :

Comitê de ética em pesquisa: Secretaria de Saúde do Estado da Paraíba

Número do parecer: 6.690.182

Data de aprovação: 7/3/2024

Certificado de apresentação de apreciação ética: 77647323.5.0000.5186

Registro do consentimento livre e esclarecido: Obtido de todos os participantes antes da coleta.

## Introdução 

Descoberto na década de 1980 (1), o vírus linfotrópico de células T humanas (HTLV), desde então, permanece subnotificado e negligenciado pelas políticas de saúde pública de diversos países. Embora seja pouco conhecida, a infecção pelo HTLV apresenta ampla distribuição geográfica e, em 2012, afetava entre 5 e 10 milhões de indivíduos em todo o mundo. Apesar da disponibilidade desses dados, a carência de estudos epidemiológicos com estatísticas mais atuais pode ter resultado em uma subestimação dos números (2). No Brasil, estimou-se que, em 2023, 1.150 milhão de pessoas estariam infectadas pelo HTLV-1, o que conferiria ao país o maior número absoluto de indivíduos infectados no mundo, com altas prevalências nas regiões Norte e Nordeste do país (3). 

O vírus é transmitido por meio de relações sexuais desprotegidas, do compartilhamento de objetos perfurocortantes contaminados e da transmissão vertical, sendo esta responsável pela manutenção da infecção entre gerações. Clinicamente, o vírus pode causar diversas manifestações clínicas que podem ter caráter neoplásico, inflamatório e infeccioso (4), com impacto direto na morbimortalidade e na qualidade de vida dos indivíduos acometidos (5). A prevalência da infecção é maior em populações vulneráveis, especialmente naquelas residentes em regiões com baixos índices de desenvolvimento humano (6).

Reconhecendo sua relevância, a infecção pelo HTLV-1 foi considerada problema de saúde pública, em especial as ações voltadas à prevenção da transmissão vertical (6). Nesse contexto de mobilização, o Brasil tem desempenhado um papel de destaque na proposição e na implementação de políticas de saúde pública voltadas à infecção, o que inclui: (i) a incorporação de testagem para HTLV no pré-natal no Sistema Único de Saúde (6-7); (ii) a meta de eliminar a transmissão vertical do HTLV como problema de Saúde Pública até 2030, como parte do programa Brasil Saudável (8); e (iii) a inclusão da infecção pelo vírus Linfotrópico de Células T Humanas, da Infecção pelo HTLV em gestante, parturiente ou puérpera e da criança exposta ao risco de transmissão vertical do HTLV na lista nacional de notificação compulsória (9). 

A resposta do Brasil ao HTLV revela-se desigual, e os estados e os municípios brasileiros encontram-se em diferentes estágios de implementação das políticas públicas preconizadas. Em algumas unidades federativas, como Bahia (10), Paraíba (11), Pernambuco (12), Mato Grosso do Sul e Distrito Federal, e em alguns municípios, como Belo Horizonte e Rio Verde, realiza-se a testagem para o HTLV no pré-natal, enquanto essa prática ainda não é uma realidade na maioria dos estados (7). 

Além desses distintos estágios de implementação, destaca-se a inexistência de um instrumento padronizado, em âmbito nacional, para a notificação compulsória dos casos confirmados da infecção pelo HTLV, bem como a ausência de diretrizes sobre o sistema de informação que deverá ser utilizado. Alguns estados, como a Bahia (10) e a Paraíba (11), já elaboraram seus próprios instrumentos de notificação, integrando-os ao Sistema de Informação de Agravos de Notificação, de âmbito nacional, e ao Sistema Estadual de Vigilância, respectivamente. No entanto, a falta de uniformidade compromete a consolidação de dados nacionais e a efetividade das ações de vigilância. Importa salientar que a notificação da infecção pelo HTLV deverá ser realizada, exclusivamente, após a confirmação diagnóstica por meio de exames confirmatórios ou moleculares (9). 

Há necessidade de construção e validação de instrumento de notificação padronizado e aplicável em todo o território nacional, contemplando os diversos ciclos de vida – incluindo crianças, gestantes, puérperas e adultos – nos serviços de saúde públicos e privados. A elaboração de tal instrumento é essencial para qualificar a vigilância epidemiológica da infecção pelo HTLV e orientar, de forma sistemática, a adoção de medidas de controle e prevenção. Este estudo teve como objetivos construir e validar um instrumento de notificação de casos da infecção pelo HTLV.

## Métodos 

### Delineamento do estudo

Trata-se de estudo metodológico, que tem como objetivos construir e validar um instrumento confiável e replicável destinado à notificação de casos de infecção pelo vírus linfotrópico de células T humanas. Tal estudo é voltado à criação de ferramentas metodológicas para coleta, organização e análise de dados, com vistas à aplicação futura em contextos similares (13). 

### Contexto 

A validação de conteúdo constituiu etapa fundamental na construção do instrumento, baseada na análise de concordância acerca dos itens e dos domínios representativos do instrumento, conforme avaliação consensual dos especialistas (14). O estudo foi conduzido em três etapas sequenciais: (i) elaboração do instrumento, a partir de revisão integrativa da literatura; (ii) validação de conteúdo por juízes especialistas com reconhecida atuação na temática HTLV; e (iii) validação de conteúdo por profissionais de saúde com experiência em vigilância epidemiológica e no enfrentamento das infecções sexualmente transmissíveis.

O desenvolvimento do instrumento baseou-se nos resultados da revisão da literatura, visando identificar evidências científicas que sustentassem sua elaboração. Para a validação de conteúdo, foi utilizada a técnica Delphi, aplicada por meio de questionários enviados a um grupo de especialistas, com o objetivo de alcançar consenso sobre a pertinência, clareza e relevância dos itens do instrumento (15). O consenso foi obtido após análise criteriosa das opiniões e justificativas dos juízes, realizadas ao longo de rodadas sucessivas de avaliação (15-16).

### Participantes 

A validação de conteúdo foi composta de duas etapas. Cada uma delas teve duas rodadas para que o instrumento pudesse ser mais bem avaliado quanto às alterações sugeridas pelos juízes. A primeira etapa contou com a participação de sete juízes especialistas, os quais tinham expertise científica com a temática do HTLV. Os critérios de elegibilidade desses juízes foram: (i) possuir especialização *lato sensu*; e (ii) ter, no mínimo, um ano de experiência científica com o HTLV. O recrutamento dos juízes ocorreu por meio de amostragem por conveniência, através de um grupo no WhatsApp composto por especialistas na temática.

A segunda etapa de validação de conteúdo teve a participação de seis juízes profissionais de saúde com experiência laboral nos programas de infecções sexualmente transmissíveis e na vigilância epidemiológica, de âmbito municipal ou estadual. Foram utilizados como critérios de elegibilidade para esses juízes: (i) atuar profissionalmente nos programas estaduais ou municipais de infecções sexualmente transmissíveis ou atuar na vigilância epidemiológica municipal e estadual; e (ii) atuar profissionalmente nesses serviços por um período mínimo de um ano. Esses especialistas foram recrutados por e-mail, com base em uma lista institucional do Ministério da Saúde.

Nas duas etapas, foi disponibilizado o registro de consentimento livre e esclarecido, por e-mail. Após a concordância em participar voluntariamente do estudo, um segundo e-mail foi enviado com o link do instrumento digitado no Google Forms, com a ficha de notificação e com uma matriz com finalidades e descrições dos itens, ambos em PDF. Foi estabelecido o prazo de 10 dias para cada juiz realizar a validação de conteúdo do instrumento.

### Variáveis 

Originalmente, o instrumento foi composto de 58 itens distribuídos em 10 categorias: dados gerais (itens: 1-9); dados sociodemográficos (itens: 10-30); provável modo de transmissão (itens: 31-33); gestante/puérpera (itens: 34-44); criança exposta (itens: 45-48); dados laboratoriais (itens 49-50); sintomatologia/doenças/infecções (itens: 51-52); evolução (itens: 53-54); observações (55); e notificante (itens: 56-58). 

Uma escala do tipo Likert, de cinco pontos, foi disponibilizada para avaliação dos itens do instrumento: 1) discordo totalmente, 2) discordo, 3) não concordo e nem discordo, 4) concordo e 5) concordo totalmente.

### Fonte de dados, mensuração 

Os dados do estudo foram coletados entre fevereiro e maio de 2024, por meio de questionário no formulário do Google Forms. Posteriormente, foram inseridos e analisados em uma planilha Excel. 

### Métodos estatísticos

Empregou-se estatística descritiva, com cálculo de números absolutos e percentuais dos dados contínuos e categóricos para a análise das variáveis sociodemográficas sobre os juízes profissionais de saúde e especialistas participantes da pesquisa. A técnica Delphi foi utilizada para a validação de conteúdo dos itens e domínios do instrumento. Para a análise estatística dos dados, foi utilizado o coeficiente Kappa, considerando-se satisfatório o valor de referência ≥0,75 para a análise dos itens e domínios do instrumento. Para calcular o coeficiente Kappa, utilizou-se o índice de validade de conteúdo e probabilidade de concordância universal (17). O software Statistical Package for Social Science, versão 26.0, foi utilizado para realização das análises estatística.

## Resultados 

Na primeira etapa de validação de conteúdo, foram convidados 60 juízes especialistas, dos quais 10 aceitaram participar da primeira rodada do estudo. Na segunda rodada, sete juízes especialistas, que contribuíram na primeira rodada, apresentaram a devolutiva da validação do instrumento. Para a segunda etapa de validação de conteúdo, foram convidados 42 juízes profissionais de saúde, dos quais 10 aceitaram participar da primeira rodada do estudo; destes, seis contribuíram com a segunda rodada de validação do instrumento. Um questionário foi elaborado com o intuito de caracterizar os participantes do estudo (Tabela 1). 

**Tabela 1:** Números absolutos (n) e percentuais (%) das variáveis sociodemográficas sobre os juízes profissionais de saúde e especialistas participantes da pesquisa. Natal, 2024 (n=13)

Variáveis	Profissionais de saúde				Especialistas			
	Primeira rodada		Segunda rodada		Primeira rodada		Segunda rodada	
	n	%	n	%	n	%	n	%
**Faixa etária** (anos)								
<30	0	0,0	0	0,0	2	20,0	2	28,6
31-40	4	40	2	33,3	2	20,0	2	28,6
41-50	5	50	4	66,7	1	10,0	1	14,3
>50	1	10	0	0,0	5	50,0	2	28,6
Sexo								
Masculino	2	20,0	2	33,3	3	30,0	1	14,2
Feminino	8	80,0	4	66,6	7	70,0	6	85,7
Profissão								
Enfermeiro(a)	8	80,0	5	83,3	0	0,0	0	0,0
Médica infectologista	1	10,0	0	0,0	0	0,0	0	0,0
Servidor público municipal	1	10,0	1	16,6	0	0,0	0	0,0
Médico(a), docente, pesquisador	0	0,0	0	0,0	3	30,0	2	28,5
Estudante de pós-graduação	0	0,0	0	0,0	2	20,0	1	28,5
Pesquisadora	0	0,0	0	0,0	1	10,0	1	14,2
Professor(a)/docente	0	0,0	0	0,0	3	3,0	2	28,5
**Área de atuação profissional no serviço atual** (anos)								
Ambulatório de infectologia	1	10,0	0	0,0	0	0,0	0	0,0
Programa de infecções sexualmente transmissíveis	6	60,0	5	83,3	0	0,0	0	0,0
Vigilância epidemiológica	3	30,0	1	16,6	1	10,0	0	0,0
Clínica e ensino	0	0,0	0	0,0	5	50,0	3	42,8
Clínica, ensino e pesquisa	0	0,0	0	0,0	2	20,0	2	28,5
Ensino e pesquisa	0	0,0	0	0,0	2	20,0	2	28,5
**Tempo de atuação profissional com o HTLV^a^** (anos)								
1-5	0	0,0	0	0,0	2	20,0	2	28,6
6-10	0	0,0	0	0,0	2	20,0	2	28,6
>10	0	0,0	0	0,0	6	60,0	3	42,9
**Região do Brasil de atuação profissional**								
Norte	3	30,0	2	28,5	2	20,0	2	28,5
Nordeste	5	50,0	2	58,5	5	50,0	3	42,8
Centro-Oeste	1	10,0	1	14,2	0	0,0	0	0,0
Sul	1	10,0	1	14,2	0	0,0	0	0,0
Sudeste	0	0,0	0	0,0	3	30,0	2	28,5
**Titulação acadêmica**								
Especialização	5	50,0	3	50,0	0	0,0	0	0,0
Mestrado	5	50,0	3	50,0	4	40,0	3	42,8
Doutorado	0	0,0	0	0,0	3	30,0	3	42,8
Pós-doutorado	0	0,0	0	0,0	3	30,0	1	14,2
**Possui publicações sobre o HTLV** **em periódicos indexados**								
Sim	0	0,0	0	0,0	7	70,0	2	71,4
Não	0	0,0	0	0,0	3	30,0	5	28,5
**Já participou de qualificação (palestras, congressos, capacitações, pesquisas) sobre o HTLV**								
Sim	7	70,0	3	50,0	0	-^b^	0	-^b^
Não	3	30,0	2	33,3	0	-^b^	0	-^b^
Não recorda	0	0,0	1	16,6	0	-^b^	0	-^b^
**Tinha conhecimento sobre a inclusão da infecção por HTLV na lista nacional de notificação compulsória de doenças, agravos e eventos de Saúde Pública, nos serviços de saúde públicos e privados**								
Sim	9	90,0	6	100,0	0	-^b^	0	-^b^
Não	1	10,0	0	0,0	0	-^b^	0	-^b^
**A linha de cuidado para HTLV está implantado no seu serviço atual**								
Sim	1	10,0	1	16,6	0	-^b^	0	-^b^
Não	6	60,0	3	50,0	0	-^b^	0	-^b^
Em andamento	3	30,0	2	33,3	0	-^b^	0	-^b^

^a^HTLV – vírus linfotrópico de células T humanas; ^b^Os itens 9, 10 e 11 foram especificamente direcionados aos juízes profissionais de saúde, visto que abordam questões relacionadas ao HTLV no contexto dos serviços de saúde.

A versão original do instrumento sofreu alterações, conforme sugestão dos juízes especialistas, as quais foram acatadas pelas pesquisadoras: no domínio *dados sociodemográficos*, foi incluída a opção “outro” no item 14 (orientação sexual). O domínio *modo provável de transmissão* sofreu alterações: o nome do item foi alterado para “transmissão sanguínea”, e foram acrescentadas as opções “uso de drogas injetáveis”, “tratamento/hemotransfusão de hemoderivados”, “transfusão sanguínea”, “exposição ocupacional a sangue e materiais biológicos por acidente com materiais perfurocortantes” e “transplante de órgão e/ou medula”.

Ainda no domínio sociodemográfico, o item 19 (número do cadastro de pessoa física) foi sugerido e validado apenas pelos juízes profissionais de saúde, em uma etapa posterior à validação pelos juízes especialistas e, por isso, esse item não foi validado por esses juízes.

No domínio de *criança exposta*, o item 46 (aleitamento materno) sofreu alteração nas alternativas, que passaram a conter as seguintes opções: “aleitamento materno exclusivo”, “aleitamento materno predominante”, “aleitamento materno complementado”, “aleitamento materno misto”, “aleitamento de banco de leite”, “aleitamento por outra pessoa” e “aleitamento artificial”. Também foi acrescentado o item 47, “tempo de aleitamento materno”, com as opções “menor ou igual a 3 meses” e “maior do que 3 meses”. No item 48, foi identificada a coleta do material biológico, sanguínea, para a realização do teste de reação em cadeia da polimerase.

No domínio *dados laboratoriais*, no item 49 (evidência laboratorial de infecção pelo HTLV), nas opções 1 e 2, as informações “positivo” e “negativo” foram excluídas. O título do item 50 (tipificação do subtipo) foi substituído por “identificação do tipo do vírus”. Na opção 3, a barra que separava a identificação dos tipos HTLV 1/2 pela letra “e” (HTLV 1 e 2) foi removida.

O domínio sintomatologia/doenças/infecções sofreu modificações no item 52 (doenças/infecções associadas), pois foram acrescentadas as opções de especificar qual/quais doenças/infecções associadas foram identificadas no momento do diagnóstico. As opções “leucemia e linfoma de Células T do Adulto” foram agrupadas em uma única alternativa. Também foi acrescentada a alternativa “outros” para que demais doenças e infecções pudessem ser reportadas.

O domínio evolução, item 53 (evolução do caso), sofreu alteração em todas as alternativas e passaram a ser: “sem deficiência”, “com deficiência”, “óbito atribuível à morbidade associada ao HTLV”, “óbito por outras causas”, “hospitalização atribuível à morbidade associada ao HTLV”, “hospitalização atribuível a outras causas” e “ignorado”. 

Embora o item 53 (evolução) tenha apresentado o coeficiente Kappa de 0,67, abaixo do limiar estabelecido (≥0,75), na validação realizada pelos juízes especialistas, as autoras julgaram que sua relevância para o acompanhamento dos casos de infecção pelo HTLV justificava a manutenção no instrumento.

Após as alterações sugeridas pelos juízes especialistas, o instrumento foi disponibilizado para a segunda etapa de validação com os juízes profissionais de saúde, que sugeriram as seguintes modificações: no domínio *dados sociodemográficos*, foram acrescentados os itens “número do Cadastro de Pessoas Físicas do paciente”, “região de saúde” e “distrito sanitário”. No domínio gestante/parturiente/puérpera, o item número da gestante no Sistema de Acompanhamento da Gestante foi removido, e o item 49 (evidência laboratorial do HTLV) foi renomeado para “evidência laboratorial do diagnóstico confirmatório da infecção pelo HTLV”.

No domínio sintomatologia/doença/infecção, foi acrescentada a opção “presença de infecções sexualmente transmissíveis. Qual? No instrumento original, essa informação encontrava-se no domínio *dados sociodemográficos*, notadamente no item de populações especiais (diagnóstico de infecções sexualmente transmissíveis).

Após as duas etapas de validação de conteúdo, com análises estatísticas que indicaram concordância ao valor de referência adotado (coeficiente Kappa≥0,75), o instrumento foi finalizado com 58 itens, distribuídos em 10 categorias: dados gerais (1-9); dados sociodemográficos (10-30); provável modo de transmissão (31-33); gestante/puérpera (34-44); criança exposta (45-48); dados laboratoriais (49 e 50); sintomatologia/doenças/infecções (51 e 52); evolução (53 e 54); observação (55); e notificante (56-58) (Tabela 2).

**Tabela 2:** Coeficientes Kappa (k) dos itens e dos domínios do instrumento validado para notificação de casos da infecção pelo vírus linfotrópico de células T humanas (HTLV). Natal, 2024 (n=62)

Questões	Profissionais de saúde		Especialistas	
	Item	Domínio	Item	Domínio
	k	k	k	k
**Dados gerais**				
Tipo de notificação	0,92		1,00	
Agravo	1,00		0,95	
Data da notificação	1,00		1,00	
Número da notificação	1,00		0,90	
Unidade Federativa da notificação	1,00	0,98	1,00	0,96
Município de notificação	1,00		1,00	
Unidade de saúde notificadora	1,00		1,00	
Cadastro Nacional de Estabelecimentos de Saúde	0,92		0,83	
Data do diagnóstico	1,00		1,00	
**Dados sociodemográficos**				
Nome do paciente	1,00		0,92	
Data de nascimento	1,00		1,00	
Idade	1,00		1,00	
Sexo biológico	1,00		0,95	
Orientação sexual	0,83		0,75	
Gestante	1,00		0,90	
Raça/cor da pele	1,00		0,95	
Escolaridade	1,00		0,95	
Número do cartão do Sistema Único de Saúde	0,90		0,95	
Número do Cadastro de Pessoa Física	1,00		0,00^a^	
Nome da mãe	1,00		0,83	
Unidade Federativa de residência	1,00		0,95	
Município de residência	1,00	0,97	0,95	0,87
Logradouro	1,00		0,82	
Bairro	1,00		0,82	
Código de Endereçamento Postal	0,95		0,82	
Telefone	1,00		0,82	
Zona	0,95		0,95	
País	1,00		0,95	
Ocupação	1,00		0,83	
Recebe algum benefício do governo	0,82		0,90	
**Provável modo de transmissão**				
Transmissão vertical	0,95		0,95	
Transmissão sexual	0,95	0,95	0,90	0,87
Transmissão sanguínea	0,95		0,75	
Gestante/puérpera				
Fez/faz pré-natal	0,87		0,83	
Unidade Federativa da realização do pré-natal	0,92		0,95	
Município de realização do pré-natal	1,00		0,95	
Unidade de realização do pré-natal	1,00		0,95	
Evidência laboratorial do HTLV	1,00		0,82	
Unidade Federativa do município de realização do parto	1,00	0,93	0,90	0,92
Município de realização do parto	1,00		0,95	
Serviço de saúde/local de realização do parto	1,00		0,95	
Data do parto	1,00		0,95	
Tipo de parto	1,00		0,90	
Evolução da gravidez	1,00		0,95	
**Criança exposta**				
Número da Declaração de Nascido Vivo	0,92		0,95	
Aleitamento materno	0,87		0,90	
Tempo de aleitamento materno	0,92	0,92	0,75	0,83
Realizada coleta sanguínea para PCR^b^	0,92		0,75	
**Dados laboratoriais**				
Evidência laboratorial de infecção pelo HTLV	0,87	0,94	0,83	0,81
Identificação do tipo do vírus	1,00		0,75	
**Sintomatologia/doenças/infecções**				
Sintomatologia	0,92	0,96	0,90	0,84
Doenças/infecções associadas	1,00		0,75	
**Evolução**				
Evolução do caso	0,95	0,97	0,67^c^	0,84
Data do óbito	1,00		0,95	
Observações				
Campo para observações	0,95	0,95	0,95	0,95
**Notificante**				
Nome do notificante	1,00		0,95	
Função	1,00	1,00	0,95	0,93
Assinatura	1,00		0,90	

^a^O item Cadastro de Pessoa Física foi sugerido pelos juízes profissionais de saúde, depois de a rodada com os juízes especialistas ter sido finalizada; ^b^PCR – reação em cadeia de polimerase; ^c^Por decisão das autoras, este item foi mantido no questionário.

## Discussão 

Os resultados obtidos demonstraram que a metodologia empregada foi eficaz na construção e validação de conteúdo do instrumento, evidenciado pelos elevados índices de concordância entre os juízes (coeficiente Kappa≥0,75). A validação permitiu a adequação de diferentes domínios do instrumento, notadamente os referentes a dados sociodemográficos, modo de transmissão, exposição infantil, dados laboratoriais e evolução clínica. A versão final do instrumento contou com 58 itens distribuídos em 10 domínios.

O número restrito de juízes especialistas que participaram das etapas de validação do instrumento constituiu uma limitação deste estudo. Esse aspecto se deve, principalmente, à escassez de profissionais com expertise consolidada em HTLV, uma infecção ainda negligenciada no cenário da saúde pública. Contudo, a qualificação técnica dos juízes assegurou a confiabilidade e a profundidade das contribuições. 

Outra limitação foi com relação à ausência da análise semântica do instrumento junto aos profissionais que realizam as notificações, etapa importante para garantir a clareza e a inteligibilidade dos itens e domínios propostos. A realização dessa fase foi inviabilizada por restrições operacionais e de tempo. 

Apesar disso, a redação dos itens e domínios foi cuidadosamente elaborada, com base em linguagem clara, acessível e apropriada à notificação da infecção pelo HTLV, e contou com o respaldo da literatura científica e as contribuições dos juízes participantes do estudo. O instrumento proposto não esgota todo o panorama da notificação sobre a infecção pelo HTLV e não teve a intenção de ser definitivo em sua completude. Portanto, o instrumento está sujeito à apreciação e à passível de adequação em consonância com as novas evidências científicas e demandas de dados que possam surgir à medida que a respostas ao HTLV avançam.

A alteração proposta no domínio *dados sociodemográficos*, notadamente no item “orientação sexual”, foi realizada considerando comportamentos sexuais com maior vulnerabilidade para infecção por HTLV-1 e 2, particularmente no que se refere à via de transmissão sexual (18).

A prevalência da infecção por HTLV-1 e 2 em grupos específicos, a exemplo de homens que fazem sexo com homens, como travestis e profissionais do sexo, permanecia elevada em 2021 (19-20). Foram embasadas as reformulações sugeridas no domínio *modo provável de transmissão*, particularmente no que se refere à inclusão de “uso de drogas injetáveis”, “tratamento/hemotransfusão de hemoderivados”, “transfusão sanguínea”, “exposição ocupacional a sangue e materiais biológicos por acidente com materiais perfurocortantes” e “transplante de órgão e/ou medula” (19-20).

Destaca-se também a relevância da reformulação do domínio *criança exposta*, especialmente no que tange ao aleitamento materno (item 46 e acréscimo do item 47 sobre o tempo de aleitamento materno). O aleitamento materno constitui um dos principais fatores que contribuem para a transmissão vertical do HTLV. As evidências demonstraram que, quanto maior o tempo de aleitamento, maior o risco de transmissão. No entanto, o aleitamento materno por mais de três meses já aumenta o risco de transmissão do vírus (21-23). 

Ainda considerando o domínio *criança exposta*, foi sugerida a inclusão da realização do teste de reação em cadeia de polimerase como item 48. Entre os métodos baseados na amplificação genômica, a reação em cadeia de polimerase se destaca com sensibilidade em torno de 70% e a especificidade de 100%, e possui custo menor quando comparado ao teste sorológico confirmatório mais utilizado, o Western blotting (24-26).

No domínio sintomatologia/doenças/infecções, foram realizadas alterações no item 52 (doenças/infecções associadas). No domínio evolução, o item 53 (evolução do caso) foi reformulado. Embora a maior parte das pessoas que vivem com HTLV permanece assintomática, alguns indivíduos desenvolvem doenças associadas à infecção que, em geral, apresentam morbidades graves e mortalidade (27). 

A infecção pelo HTLV-1 no início da vida é um fator de risco para a ocorrência de leucemia/linfoma de células T do adulto, com a probabilidade de desenvolvimento da doença de 7% em homens e 3% em mulheres. Com base na expectativa de vida de 75 anos de idade no Brasil, estima-se que ocorram entre 427 e 1.333 casos de leucemia/linfoma de células T do adulto por ano (28).

A criação desse instrumento de notificação representa uma contribuição inovadora, oportuna e estratégica para o fortalecimento da vigilância epidemiológica da infecção pelo HTLV, especialmente no contexto da sua inclusão na lista nacional de notificação compulsória. Seu uso poderá subsidiar políticas públicas mais eficazes, favorecer a alocação de recursos e ampliar o conhecimento sobre a real magnitude desse agravo no país.

## Data Availability

Os dados da pesquisa (as tabelas, o instrumento elaborado e o banco de dados) estão disponíveis no repositório SciELO Data (29) e no portal eduCapes (30).
